# Blunt abdominal injury resulting in a belly full of candy after a motocross accident, a case report

**DOI:** 10.1186/s12893-020-00997-0

**Published:** 2020-12-09

**Authors:** Floris B. Poelmann, Frank F. A. IJpma

**Affiliations:** Department of Trauma Surgery, University of Groningen, University Medical Center Groningen, Hanzeplein 1, 9713 GZ Groningen, The Netherlands

**Keywords:** Abdominal injury, Gastric perforations in children

## Abstract

**Background:**

Blunt traumatic gastric perforations in children are rare. Delayed diagnosis will lead to abdominal contamination and may result in morbidity and even mortality. We present a case of an adolescent who sustained blunt abdominal injury in a motocross accident and presented with remarkable hyperdense spherical shaped structures on the computed tomography (CT).

**Case presentation:**

A 15-year-old boy arrived at the emergency room with an acute abdomen after a motocross accident. A CT scan of the abdomen demonstrated free air and hyperdense round structures in the stomach, pelvic cavity and right paracolic gutter. During emergency laparotomy a traumatic gastric perforation was sutured, a splenic rupture was treated with a vicryl mesh and multiple spherical food scraps were removed from the abdomen. After surgery, the boy clarified that he had eaten a whole bag of colorful and spherical shaped candy just before the accident.

**Conclusions:**

Traumatic gastric rupture in children is rare but physicians should be aware of this diagnosis in case of blunt abdominal trauma with free air on the CT scan. Gastric contents, in this case candy, can present as hyperdense shaped structures in the abdominal cavity on the CT scan.

## Background

Blunt traumatic gastric perforations in children are rare [1]. An incidence has been described between 0.02 and 1.7% of all blunt abdominal trauma [2]. Traffic accidents are the most important cause of gastric rupture in case of blunt trauma [3]. Motor vehicle accidents account for approximately 90% of the gastric tears at this young age [1–3]. Delayed diagnosis may lead to abdominal contamination, resulting in morbidity and even mortality [2, 4–8]. We present a case of an adolescent who sustained blunt abdominal injury during a motocross accident and presented with remarkable hyperdense spherical shaped structures on the CT-abdomen.

## Case presentation

**Fig. 1:**
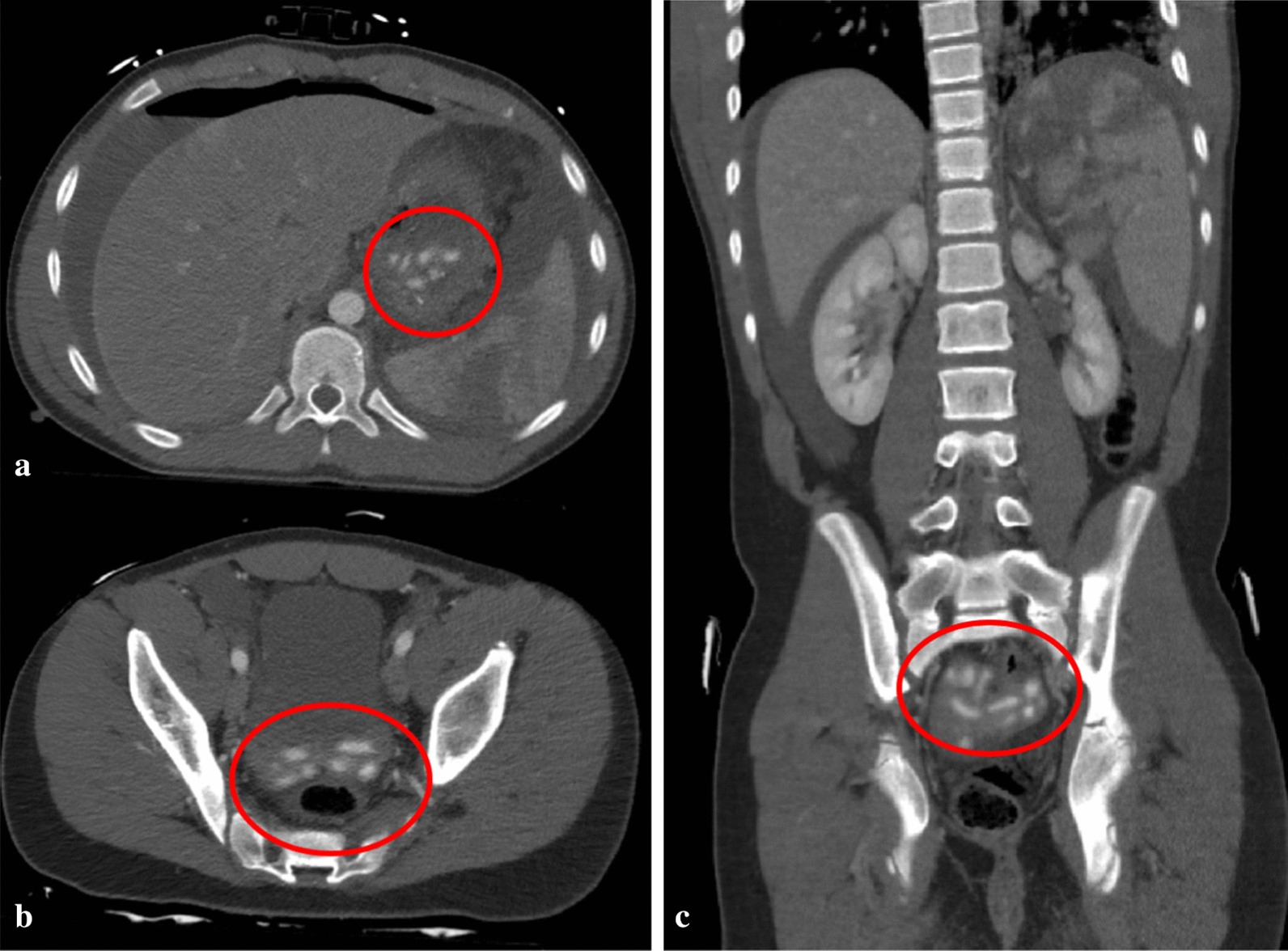
Computed tomography (CT) of the abdomen after blunt abdominal injury due to a motocross accident. **a** Axial contrast enhanced CT image of the abdomen at the portal venous phase shows free abdominal fluids, free air, a spleen laceration and remarkable spherical shaped corpora aliena in the stomach; **b** axial image, demonstrating the same hyperdense lesions in the pelvic cavity; **c** coronal image, showing a grade 4 splenic rupture as well as hyperdense lesions in the pelvic cavity

**Fig. 2:**
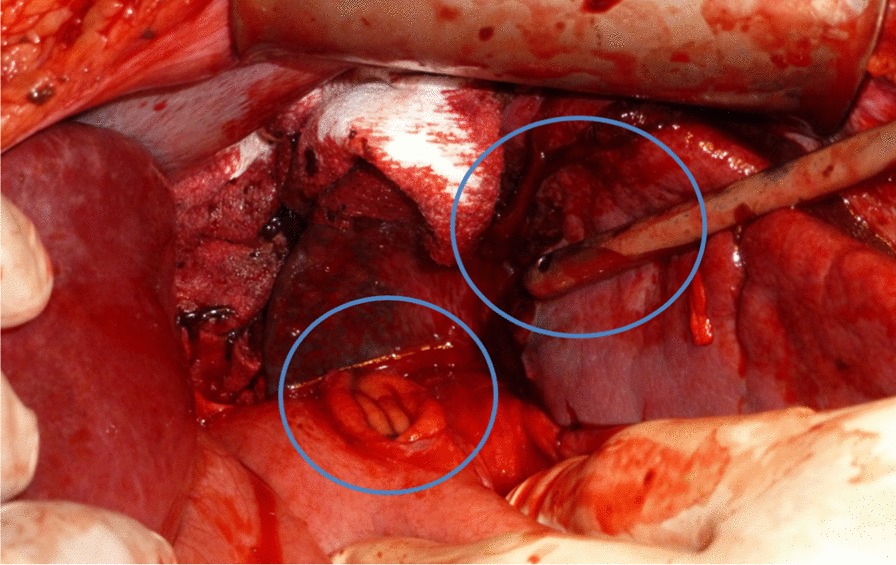
Intraoperative image of the stomach perforation (center) and a spleen laceration (center, near the tip of the suction device)

**Fig. 3:**
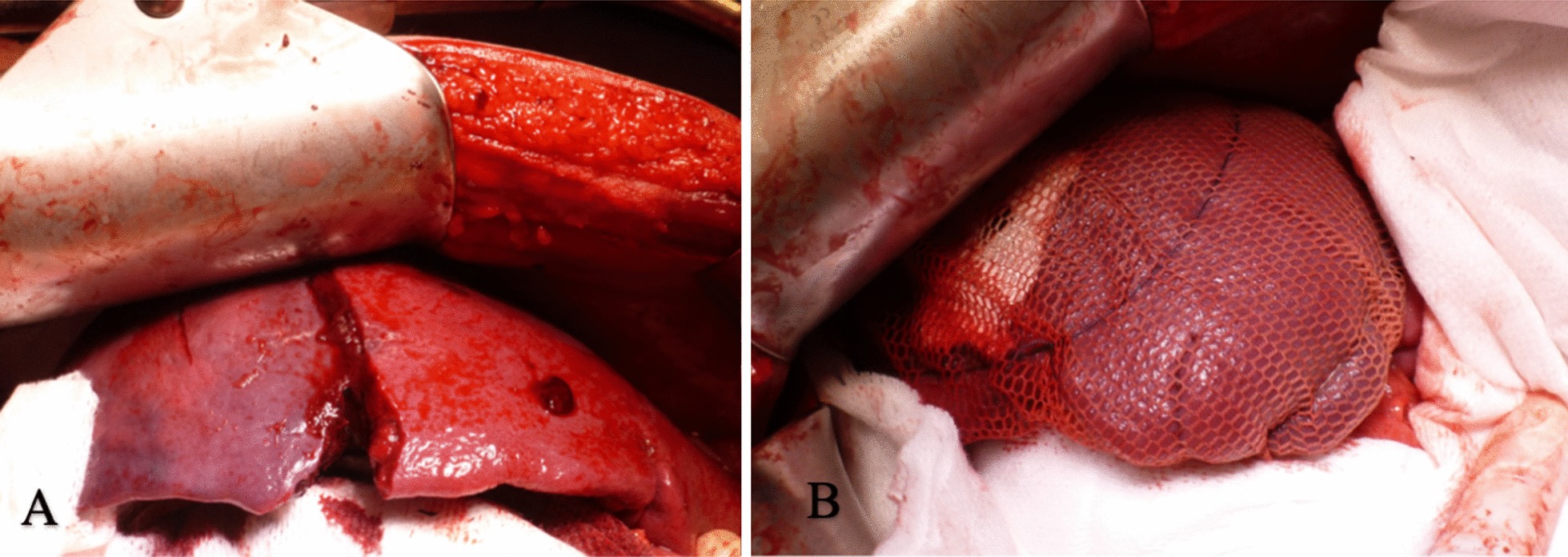
Intraoperative image of the spleen, demonstrating a spleen rupture (**a**) for which soluble vicry mesh (**b**) was placed around the injured organ

**Fig. 4:**
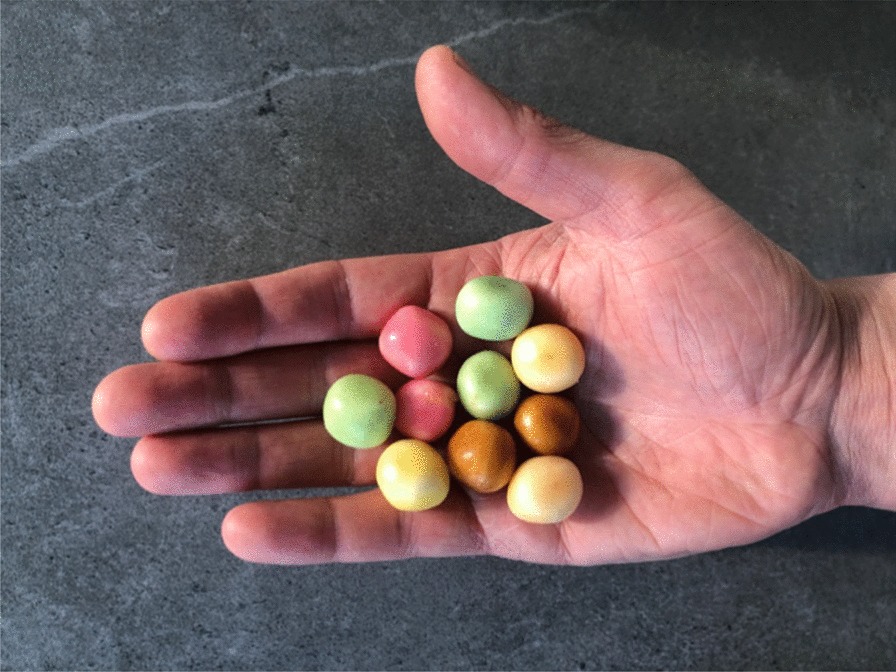
Colorful and spherical shaped candy

A 15-year-old, otherwise healthy, boy arrived at the emergency room after a motocross accident. Trauma screening was performed according to the Advanced Trauma Life Support principles. His airway was free and his cervical spine was immobilized. He had a tachypnoea of 28 breaths per minute, with normal vesicular breathing sounds and a saturation of 100%. He had a tachycardia, with 130 beats per minute, a blood pressure of 160/90 mmHg and a painful abdomen on palpation. His consciousness was slightly decreased with an EMV score of 3–5–4. X-ray of the thorax showed opacification, which was interpreted as a lung contusion. The Focused Assessment with Sonography for Trauma demonstrated free abdominal fluids. CT-angiography showed a grade IV spleen laceration and free air in the abdomen. There was no active bleeding. Also, remarkable hyperdense spherical structures were present in the stomach, pelvic cavity and right paracolic gutter (Fig. 1). Because of the presence of free air in the abdomen an emergency laparotomy was performed. During surgery, two liters of blood mixed with spherical shaped food remains were evacuated from the abdomen. A gastric perforation (diameter 5 cm) was found at the ventral side of the stomach (Fig. 2). This defect was primarily closed with sutures. The splenic rupture was treated with a Lyostipt (topical hemostatic agent). Subsequently, a soluble vircyl mesh was wrapped around the spleen in order to ensure hemostasis by compression. No further injuries were detected in the abdominal cavity (Fig. 3). After surgery, we were still unsure about the nature of the white food debris, which has been removed from the abdomen, so we asked the patient about his last meal. It appeared that he had eaten a whole bag of colorful and spherical shaped candy (Fig. 4) just before the motocross event. The patient recovered uneventfully after the operation.

## Discussion and conclusions

Traumatic gastric ruptures are rare with an estimated incidence of 0.02–1.7% of all blunt abdominal injury [2]. Gastric perforation can occur as a consequence of a high velocity impact involving the epigastric region in post-meal phase [9–11]. This trauma mechanism also applies to our case. The impact of the handlebar, striking the abdomen during the motocross accident, has probably caused a sudden increase in pressure in the full stomach, causing a gastric perforation and spreading of candy in the abdominal cavity. At the initial assessment of the CT scan, we were not yet aware that the patient had eaten a large quantity of candy. To our best knowledge, no candy-related traumatic gastric ruptures in children have been described. The hyperdense spherical structures on the CT scan, of which the nature was preoperatively unknown, appeared to be candy that entered the abdominal cavity after traumatic gastric perforation.

## Conclusion

In conclusion, traumatic gastric rupture in children is rare but physicians should be aware of this diagnosis in case of blunt abdominal trauma with free air on the CT scan. Moreover, certain released stomach contents—in this particular case candy—can present as hyperdense shaped structures at the CT scan.

## Data Availability

This case report only contains clinical data from the medical records of the patient reported herein. The data will be made available upon request.

## Abbreviation

CT: Computed tomography
